# Cyclin-Dependent Kinase 9 (CDK9) Inhibitor Atuveciclib Suppresses Intervertebral Disk Degeneration via the Inhibition of the NF-κB Signaling Pathway

**DOI:** 10.3389/fcell.2020.579658

**Published:** 2020-09-10

**Authors:** Weiyu Ni, Feizhou Zhang, Lin Zheng, Lili Wang, Yi Liang, Yuhong Ding, Jasper H. N. Yik, Dominik R. Haudenschild, Shunwu Fan, Ziang Hu

**Affiliations:** ^1^Department of Orthopaedic Surgery, Sir Run Run Shaw Hospital, Zhejiang University School of Medicine, Hangzhou, China; ^2^Key Laboratory of Musculoskeletal System Degeneration and Regeneration Translational Research, Zhejiang University School of Medicine, Hangzhou, China; ^3^The Children’s Hospital of Zhejiang University School of Medicine, Hangzhou, China; ^4^School of Statistics and Mathematics, Zhejiang Gongshang University, Hangzhou, China; ^5^Department of Orthopaedic Surgery, UC Davis Medical Center, Sacramento, CA, United States

**Keywords:** intervertebral disk degeneration, nucleus pulposus, CDK9, extracellular matrix, atuveciclib, *ex vivo*

## Abstract

Intervertebral disk degeneration (IVDD) is a spinal disk condition caused by an inflammatory response induced by various proinflammatory cytokines, such as interleukin (IL)-1β and tumor necrosis factor (TNF)-α. cyclin-dependent kinase 9 (CDK9) is a transcriptional regulator and potential therapeutic target for many diseases, especially in regulating the activation of primary inflammatory response genes. Our study investigated a highly selective CDK9 inhibitor, atuveciclib, which protects nucleus pulposus (NP) cells from proinflammatory stimuli-induced catabolism. The effects of CDK9 inhibition were determined in human and rat NP cells treated with IL-1β in the presence or absence of atuveciclib or small interfering RNA target CDK9. Inhibition of CDK9 led to the attenuation of inflammatory response. In addition, rat intervertebral disk (IVD) explants were used to determine the role of CDK9 inhibition in extracellular matrix degradation. The rat IVDD model also proved that CDK9 inhibition attenuated IVDD, as validated using magnetic resonance imaging and immunohistochemistry. Taken together, CDK9 is a potential therapeutic target to prevent IVDD.

## Introduction

Low back pain, which is one of the most common injuries and the leading cause of disability worldwide, has traditionally been thought to be an age-related degeneration of the disk tissue (Hoy et al., 2014; Foster et al., 2018). The intervertebral disk (IVD) is a special component of the spinal column that plays an important role in spinal movement (Yang et al., 2020). IVD degeneration (IVDD) is characterized by a homeostatic imbalance between anabolism and catabolism, including extracellular matrix (ECM) degradation and nucleus pulposus (NP) cell death, eventually leading to endplate sclerosis, loss of intervertebral height, and osteophyte formation (Zhao et al., 2005; Galbusera et al., 2011; Rao et al., 2016). ECM is produced by NP cells, and is the main component of the gelatinous NP tissues (Chen et al., 2018). ECM metabolism in NP cells is a dynamic process, which includes ECM anabolism and catabolism (Wang et al., 2019). Matrix metalloproteinase (MMP)-13 is reported to be one of the major catabolic factors in ECM metabolism (Le Maitre et al., 2004; MacLean et al., 2005). Aggrecan is the primary proteoglycan in the IVD and responsible for maintaining high-water content of the disk via its net negative charge that attracts cations to the ECM. Aggrecan is the most common proteoglycan, accounting for up to 50% of the NP dry weight (Walker and Anderson, 2004; Kepler et al., 2013). Both MMP-13 and aggrecan are regulated during an inflammatory response and responsible for the degradation of ECM (Luo et al., 2019). Thus, although the exact mechanism of IVDD is still not clear, inflammatory responses induced by proinflammatory cytokines are considered a primary and important cause of IVDD (Wu et al., 2018). Multiple abnormal stimuli can increase the levels of inflammatory cytokines, including interleukin (IL)-1β and tumor necrosis factor (TNF)-α, which are strongly correlated with ECM and NP cell survival (Risbud and Shapiro, 2014). These proinflammatory cytokines upregulate the generation of MMPs, and a disintegrin and metalloproteinase with thrombospondin motifs (ADAMTS) inhibit the expression of collagen 2 and aggrecan (Cheng et al., 2018). Therefore, a strategy that could effectively attenuate the inflammatory response in IVD may prevent or delay the onset of IVDD.

Primary inflammatory responses induced by acute tissue stress and inflammatory signaling do not require *de novo* protein synthesis (Yik et al., 2014). Recently, studies have reported a transcription factor, cyclin-dependent kinase (CDK) 9, which controls the expression of primary response genes by initiating transcriptional activation (Hargreaves et al., 2009; Zippo et al., 2009). In addition, CDKs belong to two partially overlapping classes: regulators of the cell cycle (CDK1, CDK2, CDK4, CDK6, and CDK7) and regulators of transcription (CDK7–CDK9 and CDK10–CDK13; Zhang et al., 2018). CDK9, a transcriptional activator, is a subunit of the positive transcription elongation factor b (P-TEFb) complex that promotes the release of paused RNA polymerase II (Pol II) promoter-proximal by phosphorylating negative elongation factors 5,6-dichlorobenzimidazone-1-ß-D-ribofuranoside (DRB sensitivity-inducing factor and negative elongation factor; Adelman and Lis, 2012). Without inflammatory signals, RNA Pol II remains at approximately 40 bp downstream of the transcription start site. During a stress response, CDK9-mediated phosphorylation of the C-terminal domain of RNA Pol II on serine 2 induces recruitment of RNA processing factors, which subsequently synthesize full-length mRNAs (Zhang et al., 2018). Thus, CDK9 may play a key role in the progression of IVDD and exert a significant impact on the activation of primary response gene transcription.

Several CDK9-targeting agents have been used for cancer therapy, such as SNS-032, dinaciclib, seliciclib, and RGB-286338. However, they lack selectivity for CDK9 and also inhibit other CDKs, resulting in treatment failure due to many adverse effects (Dai, 2003; Narita et al., 2017). The first potent and highly selective P-TEFb/CDK9 inhibitor, termed atuveciclib (BAY-1143572), has been reported and is currently undergoing clinical trials. Starting from the lead compound BAY-958, BAY-1143572 has been identified as an orally applicable CDK9-targeting candidate through a collaborative effort involving researchers from medicinal chemistry, pharmacology, drug metabolism and pharmacokinetics, structural biology, and computational chemistry (Lucking et al., 2017). It has been reported that mice treated with oral application of atuveciclib showed significantly prolonged survival compared to untreated adult T-cell leukemia/lymphoma-bearing mice (Narita et al., 2017). In addition to its potent and highly selective P-TEFb/CDK9 inhibitor, we also investigated whether atuveciclib could effectively attenuate the inflammatory response in IVDD through CDK9 inhibition.

## Materials and Methods

### Isolation and Culture of Human NP Cells

Nucleus pulposus cells were harvested from the resected specimens of patients (males, age 60 ± 20 years) with degenerative disk disease undergoing discectomy or surgery due to thoracolumbar fracture or scoliosis. The study protocol was approved by the Ethics Committee of our institution, and patients’ informed consent was obtained prior to tissue collection in accordance with the guidelines of the Ethics Committee of Sir Run Run Shaw Hospital (Zhejiang, China). NP tissue specimens were separated and washed using sterile phosphate buffered saline (PBS) three times. After cutting into pieces, NP tissues were treated with 0.25% pronase (Sigma-Aldrich, St. Louis, MO, United States) for 30 min, followed by treatment with 0.2% collagenase type II (Invitrogen, Carlsbad, CA, United States) for 4 h at 37°C. The digest was filtered through a 70 μm pore size mesh and then cultured in Dulbecco’s modified Eagle’s medium (DMEM) containing 10% fetal bovine serum (FBS; Gibco, Gaithersburg, MD, United States) in a humidified atmosphere with 5% CO_2_ at 37°C. The cultured NP cells from passages three to five were plated for all subsequent experiments.

### Harvest and Culture of Rat NP Cells

The rat NP cells were separated from the lumbar disks of Sprague Dawley rats (male, 250 g, and 8 weeks old) using a dissecting microscope and finely diced into small pieces. The samples were treated with 0.25% pronase (Sigma-Aldrich, St. Louis, MO, United States) for 30 min and digested with 0.2% collagenase type II (Invitrogen, Carlsbad, CA, United States) for 4 h at 37°C. After filtration through a 70 μm pore size mesh, rat NP cells were cultured in DMEM and Ham’s F-12 medium (DMEM/F12) supplemented with 10% FBS (Gibco, Gaithersburg, MD, United States) in a humidified atmosphere containing 5% CO_2_ at 37°C.

### Cytotoxicity Assay

The cytotoxic effects of atuveciclib were determined using cell counting kit-8 (CCK-8; Sigma-Aldrich, St. Louis, MO, United States). NP cells were seeded onto 96-well plates (2 × 10^4^ cells per well) in triplicate, and cultured in 100 μL complete DMEM or DMEM/F12 in the presence of different concentrations of atuveciclib (50, 100, 200, 400, and 800 nM) for 48 or 72 h. After washing three times with PBS, 10 μL of CCK-8 buffer was added to each well, and plates were incubated for an additional 2 h. Optical density was then measured at a wavelength of 450 nm (650 nm reference) using an ELX800 absorbance microplate reader (BioTek Instruments, Winooski, VT, United States).

### Inhibition of CDK9

Human and rat NP cells were seeded onto six-well plates (1 × 10^5^ cells per well). Atuveciclib was purchased from Selleck Chemicals (Shanghai, China) and dissolved in dimethyl sulfoxide (DMSO; Sigma-Aldrich, St. Louis, MO, United States). Cells were then treated with 200 nM atuveciclib for 48 h. CDK9 expression was knocked down using small interfering RNA (siRNA) oligonucleotides (RiboBio, Guangzhou, China). siRNAs were transfected into NP cells using Lipofectamine iMax (Invitrogen, Carlsbad, CA, United States; 1 μL/10^5^ cells). After 48 h, NP cells were collected for protein or mRNA detection. The sequence of si-CDK9 is listed in Supplementary Table S2.

### RNA Extraction and Quantitative Reverse Transcription-PCR (qRT-PCR)

Total RNA was extracted using the Ultrapure RNA kit (CWBIO, Beijing, China) according to the manufacturer’s instructions. RNA was reverse-transcribed using a HiFiScript cDNA kit (CWBio, Beijing, China). The cDNA was used to perform qRT-PCR on a 7500 Sequence Detection System (ABI, Foster City, CA, United States) using Hieff qPCR SYBR Green Master Mix (Yeason, Shanghai, China). Relative gene expression was calculated using the comparative threshold cycle method (2^–Δ^ ^Δ^ ^Ct^). Individual gene expression was normalized to that of β-actin. All primers used are listed in Supplementary Table S2.

### Western Blotting

Nucleus pulposus cells were seeded onto six-well plates (1 × 10^5^ cells per well), exposed to 10 ng/mL IL-1β (Peprotech, Beijing, China), and treated with or without 200 nM atuveciclib or transfected with si-CDK9. To determine the effect of atuveciclib on signaling pathways, NP cells were seeded onto six-well plates at a density of 1 × 10^5^ cells/well. The cells were pretreated with 200 nM atuveciclib for 2 h. Untreated cells were used as controls. Next, cells were stimulated with 10 ng/mL IL-1β for 0, 5, 10, 20, 30, or 60 min. NP cells were then lysed using RIPA lysis buffer (CWBio, Beijing, China), and total proteins were quantified using a bicinchoninic acid analysis kit (Beyotime, Shanghai, China). Proteins (20 μg per well) were separated on 10% sodium dodecyl sulfate-polyacrylamide gels and then transferred onto polyvinylidene difluoride membranes (Bio-Rad, Hercules, CA, United States). The membranes were blocked in 5% skim milk in Tris–HCl buffer containing 0.1% Tween-20 at room temperature for 1 h, and then incubated with primary antibodies specific for MMP-3, MMP-13, ADAMTS5, collagen 2 (all at 1:1000 dilution; Abcam, United States), aggrecan (1:1000 dilution; ABclonal, China), CDK9, extracellular regulated protein kinases (ERK), phosphorylated (p)-ERK, p65, p-p65, IκBα, p-IκBα, and β-actin (all at 1:1000 dilution; Cell Signaling Technology, United States) overnight at 4°C. Protein bands were developed using a horseradish peroxidase-conjugated immunoglobulin (1:5000; Cell Signaling Technology, United States) at room temperature for 1 h, followed by detection using ECL reagent (Fudebio, Hangzhou, China). Protein bands were visualized using the LAS-4000 Science Imaging System (Fujifilm, Tokyo, Japan).

### Immunofluorescence (IF)

Human NP cells were seeded on coverslips, exposed to 10 ng/mL IL-1β, and treated with or without 200 nM atuveciclib or transfected with si-CDK9. After 1 or 48 h, cells were fixed with 4% paraformaldehyde for 10 min and washed three times with PBS, followed by permeabilization using 0.1% Triton-X100 for 10 min at room temperature. NP cells were washed three times with PBS again and blocked in 10% goat serum for 1 h before incubation with MMP-13, ADAMTS5, aggrecan, and collagen 2 antibodies (1:200; Abcam) at 4°C overnight. After washing three times with PBS, samples were incubated with Cy5-conjugated goat anti-rabbit IgG (1:200; Abcam) in PBS for 1 h, and then stained with DAPI for 10 min at room temperature (Life Technologies, Carlsbad, CA, United States). Fluorescence signals were imaged using a fluorescence microscope (MODEL BX51TRF; Olympus, Tokyo, Japan).

### Alcian Blue and Toluidine Blue Staining

Nucleus pulposus cells were seeded onto six-well plates (1 × 10^5^ cells per well), exposed to 10 ng/mL IL-1β, and treated with or without 200 nM atuveciclib or transfected with si-CDK9. After 7 days, NP cells were fixed with 4% paraformaldehyde for 20 min and stained with 1% toluidine blue (Sigma-Aldrich, St. Louis, MO, United States) for 10 min. The Alcian blue standard staining kit (Solarbio, Beijing, China) was used for histological staining of the NP cells. Briefly, samples were treated with Alcian acidizing buffer for 5 min and then stained with Alcian staining buffer for 30 min. Relative values of proteoglycans (PGs) were determined by measuring the intensity of Alcian blue staining or toluidine blue staining using ImageJ (1.48, NIH, Bethesda, MD, United States).

### Isolation and Culture of IVD Specimens

*Ex vivo* experiments were performed as previously described (Chen et al., 2016). Lumbar IVDs, including the adjacent vertebral endplates, were isolated from 10 Sprague Dawley rats (male, 250 g, and 8 weeks old). Specimens were washed in Hank’s solution (Solarbio, Beijing, China) containing 55 mM Na-citrate (Sigma-Aldrich). The IVDs were washed with agitation overnight in DMEM supplemented with 5% fetal calf serum and 20 mM Na-citrate in an incubator (37°C, 5% CO_2_). IVD specimens were then exposed to 100 ng/mL IL-1β and treated with or without 200 nM atuveciclib for up to 1 week on 48-well plates containing DMEM supplemented with 10% fetal calf serum and 25 g/mL L-ascorbate.

### Co-immunoprecipitation (co-IP) Assay

Nucleus pulposus cells were seeded in 6 cm dishes (5 × 10^5^ cells per well), exposed to 10 ng/mL IL-1β, and treated with or without 200 nM atuveciclib for 1 h. Protein A/G MagBeads (Yeason, Shanghai, China) were incubated with anti-p65 or anti-CDK9 antibody (10 μg/mL; Abcam) diluted with binding buffer (50 mM Tris, 150 mM NaCl, 0.1% Tween 20, pH 7.5) at room temperature for 15 min. The cells were washed, lysed, and collected according to the manufacturer’s protocol. After washing three times using a buffer solution (50 mM Tris, 150 mM NaCl, 0.5% Tween 20, pH 7.5), the beads were collected and incubated with the cell extracts at 4°C overnight. Next, Protein A/G MagBeads-antigen-antibody complexes were collected and washed using the same washing buffer. Bound proteins were resolved using an elution buffer (0.1 M glycine, 0.1% Tween 20, pH 3.0), followed by a western blotting assay, as described above.

### A Rat Model of IVDD

All animal protocols were performed in accordance with standard ethical guidelines as approved by the Ethics Committee of Sir Run Run Shaw Hospital (Zhejiang, China). A total of 24 male Sprague Dawley rats, aged 12 weeks, were purchased from Shanghai SLAC Laboratory Animal, Co., Ltd. (Shanghai, China). The rats were placed in a prone position after being anesthetized by administering intraperitoneal injection of 90 mg/kg ketamine and 10 mg/kg xylazine. Under fluoroscopic guidance, the tail skins of 12 rats were was disinfected with ethanol and punctured using an 18-gauge needle from the dorsal side at the same disk. The needle was punctured through the center of the disk until the opposite side, rotated 180°, and held for 10 s. The remaining 12 rats underwent no surgical intervention and served as the negative controls. Post-surgery, the wound was covered with gauze and a standard postoperative procedure was performed. After 8 weeks, the rats were euthanized using an overdose of chloral hydrate.

### Atuveciclib Injection

The small molecule CDK9 inhibitor molecule, atuveciclib, was dissolved in DMSO to achieve a stock concentration of 10 mg/mL. Atuveciclib (10 mg/kg) diluted in sterile saline was administered intraperitoneally via a 30-gauge needle immediately after animal surgery. Thereafter, the drug was administered daily for up to 14 days for the IVDD model, or three times weekly for up to 8 weeks for the ovariectomy model. The negative controls (untreated animals) received only saline injections.

### Magnetic Resonance Imaging (MRI)

Magnetic resonance imaging (MRI) scans were performed 8 weeks after surgery. The water content of lumbar IVD was measured on sagittal T2W1 MRI. IVD height was measured using the ImageJ software and expressed as the disk height index (DHI) using a previously described method (Masuda et al., 2005). The MRI parameters were set according to a previous report (Chen et al., 2020) namely, slew rate at 150 mT/m/ms and gradient field intensity of 30 mT/m. Parameters for spin-echo sequence were T2W1/TR, TE = 3500 ms/120 ms, scan matrix: 256 × 256, reconstruction matrix: 512 × 512, FOV (mm) = 100.00, RFOV (%) = 100.00, slice thickness = 3 mm, and scan resolution = 0.3 mm.

### Histological Analysis

Harvested disks were fixed in 4% buffered paraformaldehyde, decalcified in 10% ethylene diamine tetraacetic acid (EDTA) for 1 month, embedded in paraffin, and sectioned at 4 μm thickness. Histological sections were stained with 0.1% Safranin O and 0.001% Fast Green solution, or Alcian blue solution (pH 2.5) to reveal morphology and matrix degeneration. For evaluation using immunohistochemistry (IHC), slides were incubated with sodium citrate antigen retrieval solution (Solarbio, Beijing, China) at 60°C overnight. Hydrogen peroxide (3%) was used to block endogenous peroxidase activity. Thereafter, rat disks were blocked by incubation with 5% (w/v) bovine serum albumin and incubated with primary antibodies against collagen 2 and aggrecan (at 1:200 dilution; Abcam) at 4°C overnight. Histological images were captured using a BX53 microscope (CX33TRF; Olympus, Tokyo, Japan).

### Statistical Analysis

All data were presented as the mean ± standard deviation. Data analyses were performed using SPSS 19.0 (SPSS, Chicago, IL, United States). Statistical differences were analyzed using one-way analysis of variance or Student’s *t*-test. Results were considered statistically significant at *P* ≤ 0.05.

## Results

### Atuveciclib Is a Potent and Highly Selective CDK9 Inhibitor

**FIGURE 1:**
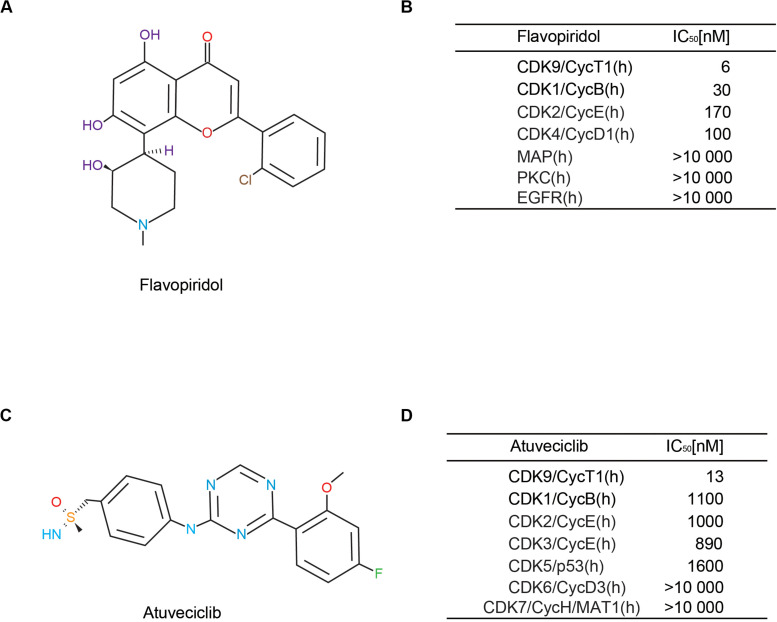
Comparison of different cyclin-dependent kinase 9 (CDK9) inhibitors. **(A,B)** Structure and CDK-inhibitory activities of flavopiridol. **(C,D)** Structure and CDK-inhibitory activities of atuveciclib (BAY 1143572).

Based on our previous study, flavopiridol (Figure 1A) is an adenosine triphosphate (ATP) analog that preferentially inhibits CDK9 activity, and is the first non-selective CDK inhibitor to be used clinically (Yik et al., 2014; Hu et al., 2018). It possesses high inhibitory activity against CDK9, with a half-maximal inhibitory concentration (IC_50_) of 6 nM. Flavopiridol has also been shown to inhibit the activities of CDK1, CDK2, CDK4, CDK6, CDK7, and CDK9 (Figure 1B). Recently, drug researchers have been focusing on the aspect of high selectivity with respect to the CDK family, particularly, high CDK9 specificity. Here, we demonstrated that atuveciclib, a new selective CDK9 inhibitor (IC_50_ CDK9/CycT1: 13 nM, ratio of IC_50_ values CDK2/CDK9: 100), exhibited the best overall inhibition profile *in vitro* and *in vivo* (Figures 1C,D). In addition, an *in vivo* pharmacokinetic study reported that atuveciclib demonstrated low blood clearance (Lucking et al., 2017). Therefore, atuveciclib was exclusively employed as a CDK9 inhibitor in this study.

### Atuveciclib Treatment Attenuated IL-1β-Induced IVDD

**FIGURE 2:**
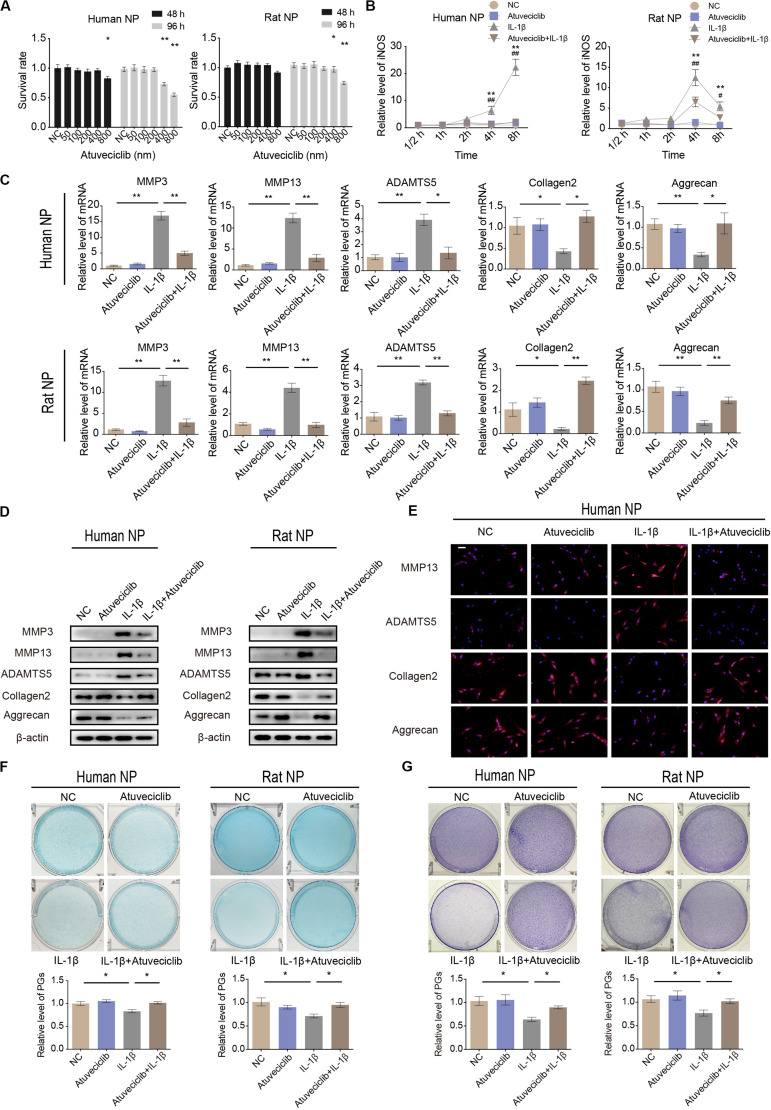
Atuveciclib inhibits extracellular matrix (ECM) degradation in human and rat nucleus pulposus (NP) cells. **(A)** Cytotoxic effects of atuveciclib on human and rat NP cells were assessed using cell counting kit-8 (CCK-8) cell viability and toxicity assays at 48 and 96 h. **(B)** Time-dependent suppression of atuveciclib on inducible nitric oxide synthase (iNOS) expression. Human and rat NP cells were treated with interleukin (IL)-1β and indicated concentrations of atuveciclib to determine the time response for suppressing iNOS induction. *denotes statistical significance between IL-1β-treated and control groups, ^#^denotes statistical significance between IL-1β-treated and IL-1β + atuveciclib-treated groups. **(C,D)** qRT-PCR **(C)** and western **(D)** blot analysis of matrix metalloproteinase (MMP)-3, MMP-13, ADAMTS5, aggrecan, and collagen 2 levels in human and rat NP cells treated with IL-1β and atuveciclib for 48 h. **(E)** IF analysis of MMP-13, ADAMTS5, aggrecan, and collagen 2 in human NP cells treated with IL-1β and atuveciclib for 48 h. **(F,G)** Human and rat NP cells were treated with IL-1β and atuveciclib for 7 days. The total amount of proteoglycans (PGs) released in the culture medium was measured using Alcian blue **(F)** and toluidine blue staining **(G)**. Data are representative images among similar results obtained from three different donors **(D–G)** or presented as the mean ± SEM of three independent experiments **(A–C)**. *P < 0.05, **P < 0.01 vs. control, ^#^*P* < 0.05, ^##^*P* < 0.01, as indicated by Student’s *t*-test.

To investigate the potential cytotoxicity of atuveciclib, cell viability assay was performed. No cytotoxicity was observed at a dose of 200 nM in both human and rat NP cells at 48 and 96 h (Figure 2A). Since IL-1β activates the primary inflammatory response genes and induces IVDD, NP cells were treated with IL-1β in the presence or absence of atuveciclib. The expression of the stress response gene, inducible nitric oxide synthase (iNOS), was determined at various time points. The mRNA level of iNOS increased significantly at 4 h post treatment with IL-1β in both human and rat NP cells. However, co-treatment with atuveciclib significantly suppressed the expression of iNOS in human NP cells and partly attenuated in rat NP cells (Figure 2B). To further confirm the effects of atuveciclib on inflammatory response, NP cells were co-treated with IL-1β and atuveciclib for 48 h. IL-1β stimulation increased the mRNA and protein levels of MMP-3, MMP-13, and ADAMTS5 and decreased the levels of aggrecan and collagen 2; however, these effects were significantly attenuated using treatment with atuveciclib in both human and rat NP cells (Figures 2C,D). IF assay performed in human NP cells further confirmed these results (Figure 2E). Furthermore, NP cells were stained with Alcian blue and toluidine blue to determine the distribution of PGs. After treatment with IL-1β, the levels of PGs were decreased, and co-treatment with atuveciclib significantly suppressed this effect (Figures 2F,G). Taken together, atuveciclib effectively attenuated the inflammatory response stimulated by IL-1β, thus confirming its role as a potent CDK9 inhibitor.

### Attenuation of Inflammatory Response by CDK-9 Knockdown in NP Cells

**FIGURE 3:**
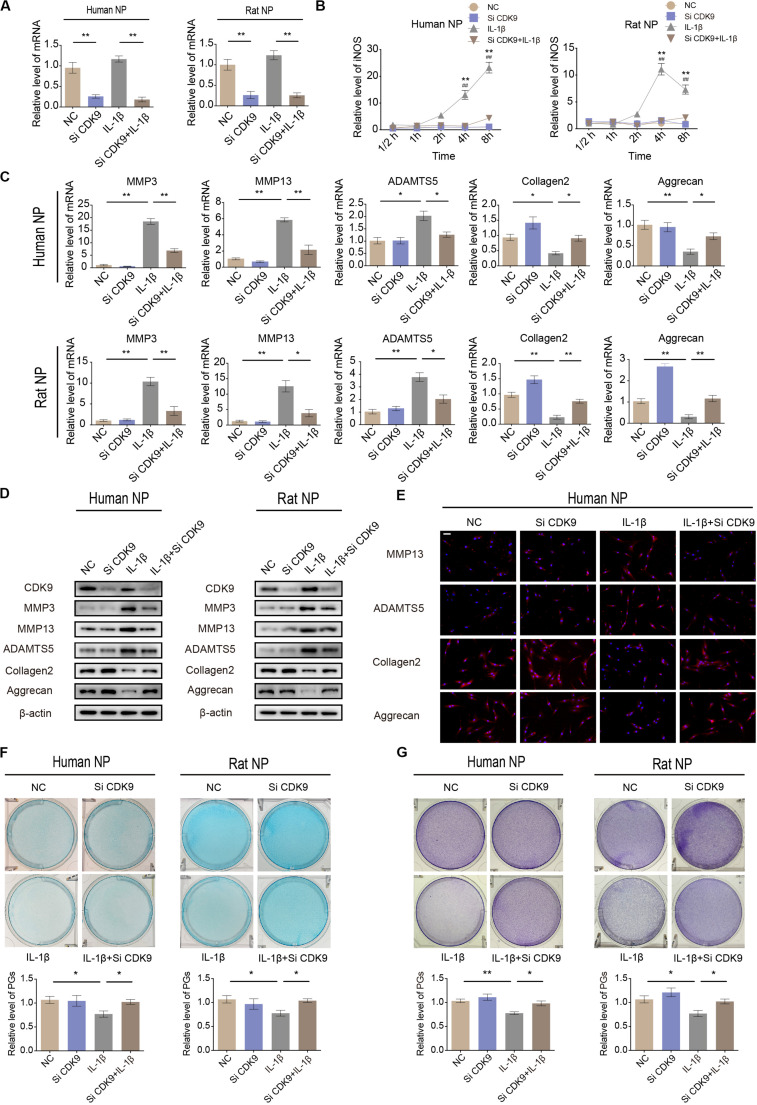
Knockdown of cyclin-dependent kinase 9 (CDK-9) attenuates extracellular matrix (ECM) degradation in human and rat nucleus pulposus (NP) cells. **(A)** Relative expression of CDK9 in human and rat NP cells treated with negative control small interfering RNA (siRNA) or CDK9 siRNA and stimulated with interleukin (IL)-1β. **(B)** Time-dependent suppression of inducible nitric oxide synthase (iNOS) induction using siRNA. Human and rat NP cells were treated with IL-1β and transfected with CDK9 siRNA to determine the time response for suppressing iNOS induction. *denotes statistical significance between IL-1β-treated and control groups, ^#^denotes statistical significance between IL-1β-treated and IL-1β + atuveciclib-treated groups. **(C,D)** Human and rat NP cells were transfected with CDK9 or negative control siRNA, and then exposed to IL-1β for 48 h. qRT-PCR **(C)** and western blotting **(D)** analyses were performed for matrix metalloproteinase (MMP)-3, MMP-13, ADAMTS5, aggrecan, and collagen 2 detection. **(E)** IF analysis was used to determine the expression of MMP-13, ADAMTS5, aggrecan, and collagen 2 in human NP cells transfected with CDK9 or negative control siRNA, and then exposed to IL-1β for 48 h. **(F,G)** Human and rat NP cells were treated with IL-1β and transfected with CDK9 siRNA for 7 days. The total amount of proteoglycans (PGs) released in the culture medium was measured using Alcian blue **(F)** and toluidine blue staining **(G)**. Data are representative images among similar results obtained from three different donors **(D–G)** or presented as the mean ± SEM of three independent experiments **(A–C)**. *P < 0.05, **P < 0.01 vs. control, ^#^*P* < 0.05, ^##^*P* < 0.01, as indicated by Student’s *t*-test.

To identify the role of CDK9 in IVDD, human and rat NP cells were transfected with CDK9-specific siRNAs. The efficiency of siRNA transfection was confirmed using qRT-PCR (Figure 3A). qRT-PCR results revealed that silencing of CDK9 inhibited the expression of iNOS, indicating that the stress response gene was not fully activated without CDK9 (Figure 3B). In NP cells, IL-1β treatment enhanced the expression of ECM-degrading enzymes but inhibited ECM protein expression. Notably, knockdown of CDK9 was remarkably impaired by IL-1β, as assessed using qRT-PCR, western blotting, and IF (Figures 3C–E). In addition, after co-treatment with IL-1β and si-CDK9, NP cells resumed secreting normal levels of PGs, as indicated in the results of Alcian blue and toluidine blue staining (Figures 3F,G). Thus, our findings reveal an important role of CDK9 in IVDD *in vitro*.

### Atuveciclib Attenuates Matrix Degradation in an *ex vivo* Model

**FIGURE 4:**
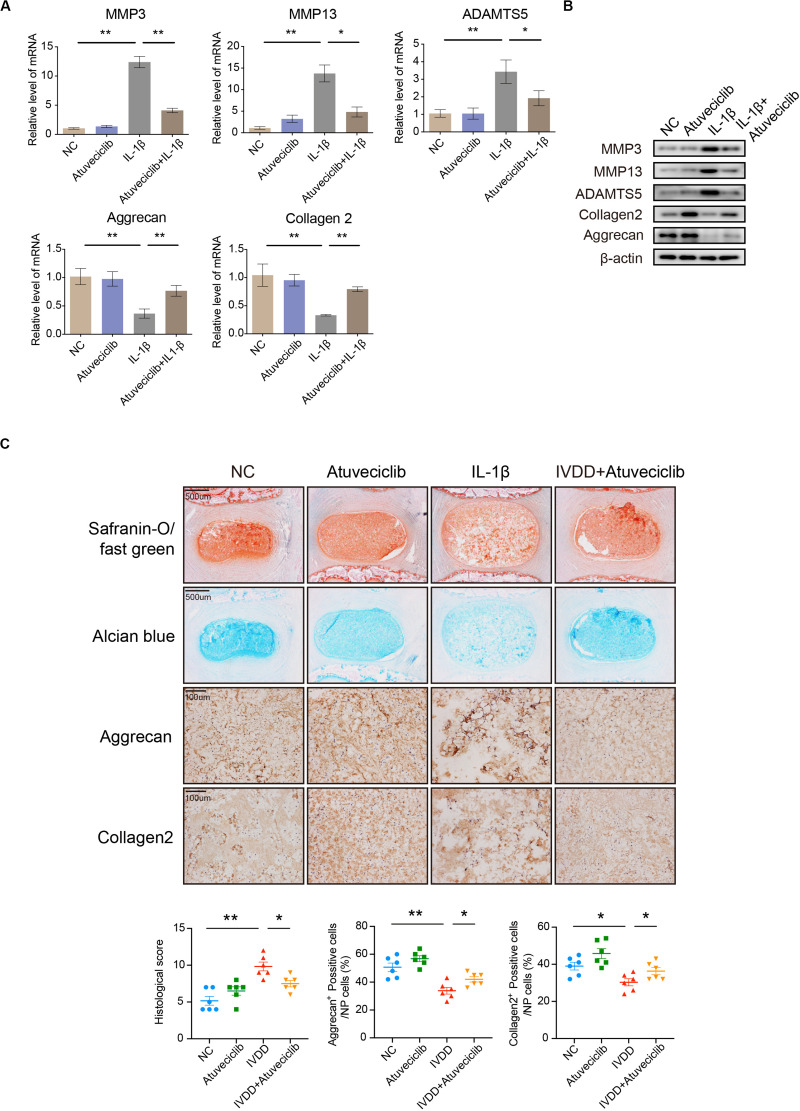
The role of atuveciclib in an *ex vivo* model. **(A,B)** mRNA **(A)** and protein **(B)** levels of matrix metalloproteinase (MMP)-13, ADAMTS5, aggrecan, and collagen 2 in rat disks, which were exposed to 100 ng/mL interleukin (IL)-1β and treated with or without 200 nM atuveciclib for up to 1 week in an *ex vivo* model. **(C)** Safranin O and Fast green staining, Alcian blue staining, and immunohistochemistry (IHC) assay of disk sections treated with or without IL-1β for the indicated times. Histological scores of the different groups were determined using Safranin-O/Fast green staining. Data are representative images among similar results obtained from three different donors **(B,C)** or presented as the mean ± SEM of three independent experiments **(A)**. *P < 0.05, **P < 0.01 vs. control, as analyzed using Student’s *t*-test.

Since inflammatory response was partially attenuated by using atuveciclib treatment in NP cells, we next performed experiments to confirm the effects of atuveciclib in a whole disk *ex vivo* model. RNA and proteins were extracted from rat IVDs exposed to IL-1β with or without atuveciclib treatment. As expected, IL-1β treatment activated the markers of catabolism (MMP-3, MMP-13, and ADAMTS5) and inhibited those of anabolism (collagen 2 and aggrecan); however, co-treatment using atuveciclib dramatically attenuated these effects (Figures 4A,B). To further investigate matrix degradation in the *ex vivo* model, rat IVD tissues were collected and assessed histologically. Safranin O/Fast green and Alcian blue staining showed that the ECM was significantly degraded by IL-1β stimulation, and treatment with atuveciclib partly attenuated this effect (Figure 4C). Histological scores based on Safranin O and Fast green staining confirmed these results. In addition, IHC assay demonstrated that treatment with atuveciclib reversed the IL-1β-induced downregulation of aggrecan and collagen 2 expression (Figure 4C). These results demonstrate that atuveciclib could block the IL-1β-induced matrix degradation in an *ex vivo* model.

### Atuveciclib Treatment Suppressed Inflammatory Responses via Inhibition of NF-κB Pathway Activation

**FIGURE 5:**
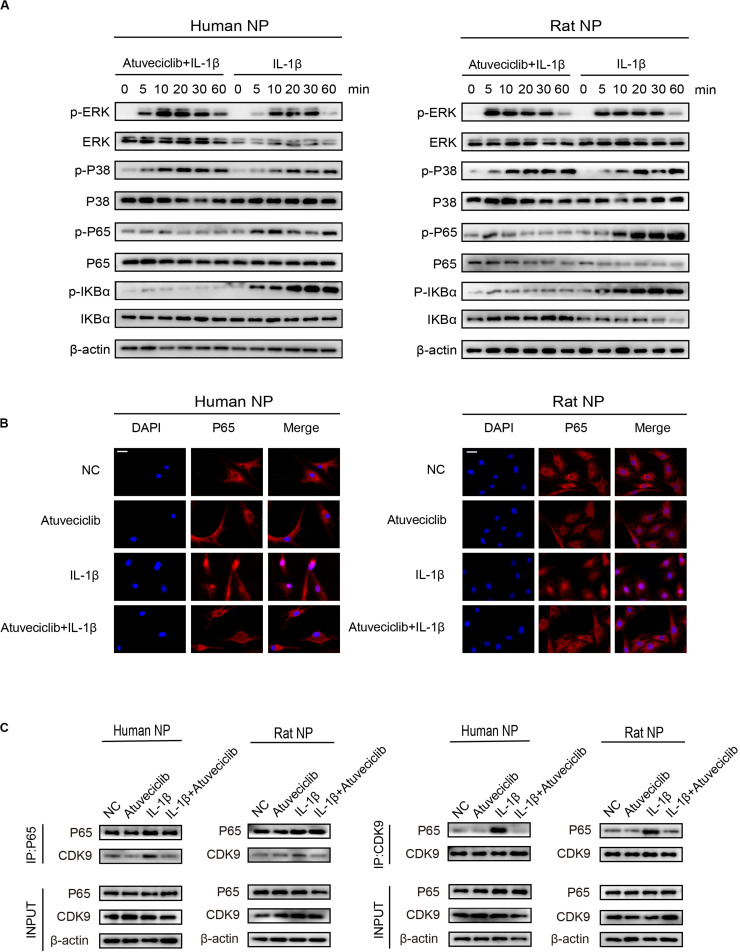
Atuveciclib inhibits the phosphorylation and nuclear translocation of P65. **(A)** Total cellular protein extracted from human and rat nucleus pulposus (NP) cells treated with IL-1β and atuveciclib for 0, 5, 10, 20, 30, or 60 min were subjected to western blot analysis using specific antibodies against extracellular regulated protein kinases (ERK), phosphorylated (p)-ERK, p38, p-p38, p65, p-p65, IKBα, and p-IKBα. **(B)** Human and rat NP cells were pretreated with atuveciclib for 2 h before stimulation using IL-1β for 30 min. Nuclear translocation of p65 was visualized using IF assays. **(C)** Human and rat NP cells were pretreated with atuveciclib for 2 h before stimulation with IL-1β for 30 min, and total cellular protein extracted were subjected to immunoprecipitation using specific antibodies against p65 and cyclin-dependent kinase 9 (CDK9). Immunoprecipitants were then subjected to western blot analysis using specific antibodies against CDK9 and p65. Data are representative images among similar results obtained from three different donors **(A–C)**.

To elucidate the role of atuveciclib in IL-1β-induced matrix degradation, we examined key signaling pathways, including the mitogen-activated protein kinases (MAPK) and NF-κB signaling pathways. A time course study was conducted using IL-1β-treated NP cells, with or without atuveciclib treatment. Western blot analysis showed that both MAPK and NF-κB pathways were activated by using IL-1β treatment. However, co-treatment using atuveciclib inhibited the phosphorylation of I-kappa-B (IKB)α and p65, and exhibited no obvious inhibitory effects on the MAPK signaling pathway (Figure 5A). Thus, we assumed that atuveciclib exerts its function by regulating the NF-κB pathway. To further determine the role of atuveciclib, we examined p65 localization after 1 h of stimulation with IL-1β. IF staining showed that atuveciclib treatment inhibited the IL-1β-induced p65 nuclear translocation (Figure 5B). A previous study revealed that NF-κB binds to P-TEFb, and consisting of CDK9, to stimulate transcriptional elongation by RNA Pol II (Barboric et al., 2001). Using an IP assay, we found that IL-1β stimulation enhanced interaction between p65 and CDK9, and atuveciclib co-treatment attenuated this effect (Figure 5C). Hence, atuveciclib was shown to be involved in the inflammatory response and regulate the expression of inflammatory factors via the suppression of p65 phosphorylation and nuclear translocation.

### Intraperitoneal Injection of Atuveciclib Alleviated IVDD in a Rat Model

**FIGURE 6:**
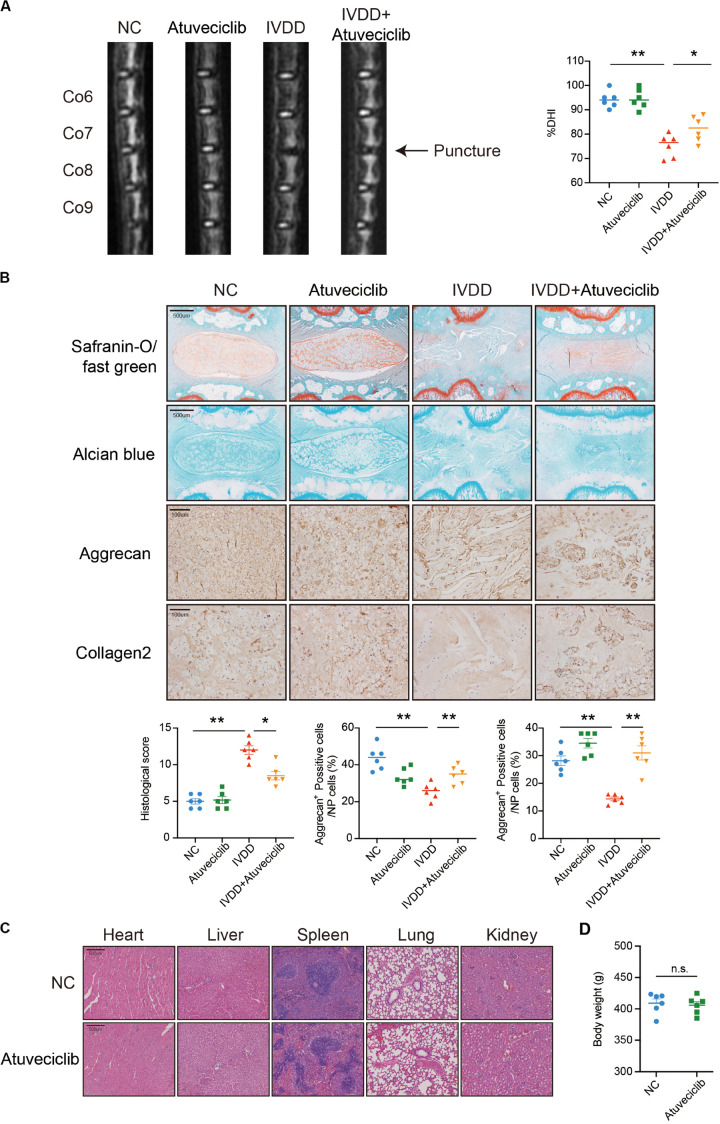
Role of atuveciclib in a rat intervertebral disk degeneration (IVDD) model. **(A)** Magnetic resonance imaging (MRI) images and disk height index (DHI) of the rat model at 8 weeks after operation. **(B)** Safranin O and Fast green staining, Alcian blue staining, and immunohistochemistry (IHC) assay of disk sections. The histological score of the different groups was assessed using Safranin-O/Fast green staining of rats at 8 weeks post operation. Histological scores of the different groups were determined using Safranin-O/Fast green staining. **(C)** Hematoxylin and eosin (H&E) staining of the heart, liver, spleen, lung, and kidney tissues of rats in the treatment and control groups. **(D)** The body weight of experimental animals of the treatment and control groups after 8 weeks. Data are representative images among similar results or presented as the mean ± SEM obtained from six different donors **(A–D)**. *P < 0.05, **P < 0.01 vs. control, n.s., not significantly different, as analyzed using Student’s *t*-test.

**FIGURE 7:**
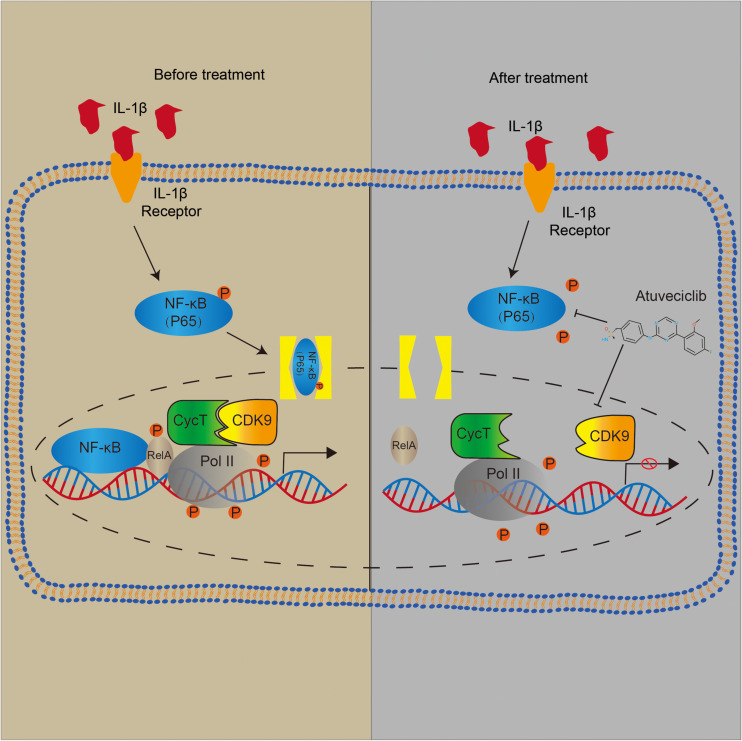
Schematic representation of major molecular pathways.

To investigate the effect of atuveciclib *in vivo*, we successfully established a rat model for IVDD using needle puncture. At 8 weeks after the puncture, with or without intraperitoneal injection of atuveciclib, IVDD was assessed using MRI. MRI images showed significantly weaker signal intensities in the IVDD group than in the control group. However, after treatment with atuveciclib, IVDD progression was suppressed (Figure 6A). To further investigate IVDD progression, Safranin O/Fast green and Alcian blue staining were performed. Following the progression of IVDD, the ECM was degraded and the levels of PGs decreased. Moreover, according to the statistical analysis on results of Safranin O and Fast green staining, the histological score of the IVDD group was significantly higher than that of the control group. Histological analysis revealed that treatment with atuveciclib partly attenuated the phenotypes of IVDD (Figure 6B). IHC staining for aggrecan and collagen 2 further confirmed these results. Furthermore, to exclude the toxicity of atuveciclib, hematoxylin and eosin (H&E) staining of the heart, liver, spleen, lung, and kidney tissues and the body weight revealed no obvious differences between the atuveciclib-injected and control groups (Figures 6C,D). Taken together, the role of atuveciclib in IVDD prevention was demonstrated using an *in vivo* model. The study design is been schematically illustrated in Figure 7.

## Discussion

At present, a number of CDK9 inhibitors are undergoing various clinical studies. Flavopiridol is a member of the first generation CDK inhibitors, which acts as a ‘pan’ CDK antagonist. To address its low selectivity for a specific CDK, more target-specific CDK inhibitors have been developed (Dai, 2003). The first CDK inhibitor developed was olomoucine (IC_50_ = 60. 3 μM), which is a selective inhibitor for CDKs 1, 2, 5, and 7 (Hardcastle et al., 2002). Another CDK inhibitor, indolinone (IC50 = 40 nM) also selectively inhibits CDKs 1, 2, and 5 (Lane et al., 2001). In addition, LY2857785 has been shown to selectively inhibit CDK9 (IC50 = 0.011 μM) by inhibiting the phosphorylation of RNA Pol II. Among all these different types of CDK9 inhibitors, atuveciclib is most notable for its potent and highly selective functions (Figure 1).

Since CDK9 plays a key role in inflammatory response, we evaluated the function of CDK9 in IL-1β-stimulated IVD cells. Results showed that treatment with IL-1β increased the expression of the stress response gene, iNOS, as well as ECM degradation. In the presence of atuveciclib, inflammatory response was significantly suppressed, and the expression of catabolic enzymes (MMP-3, MMP-13, and ADAMTS5) was downregulated, while that of components of the ECM (aggrecan and collagen 2) was upregulated. An increase in the levels of PGs further enhanced ECM restoration (Figure 2). Hence, for the first time, our study demonstrated that treatment with a CDK9 inhibitor (atuveciclib) disrupted the IL-1β-stimulated inflammatory response, which subsequently attenuated ECM degradation in NP cells under stress. ECM is produced by NP cells and is the main component of the gelatinous NP tissue (Chen et al., 2018). Inflammatory cytokines, such as IL-1β and TNF-α, stimulate the expression of ECM catabolic enzymes, but inhibit that of ECM matrix (Le Maitre et al., 2007; Wang et al., 2011). Loss of ECM has been shown to cause disk height reduction, resulting in alteration of mechanical characteristics of the spine, and eventually leading to progressive IVDD (Chen et al., 2016). Here, the significant effects of atuveciclib indicate that CDK9 plays a key role in IVDD progression. To further confirm the function of CDK9, NP cells were treated with si-CDK9 instead of atuveciclib. In both human and rat NP cells, knockdown of CDK9 attenuated the effects of IL-1β stimulation (Figure 3). To simulate the internal environment, rat IVD specimens were isolated and cultured for co-treatment with atuveciclib and IL-1β. In our *ex vivo* studies, we found that IL-1β treatment increased anabolism and decreased catabolism, and this effect was partially blocked by using atuveciclib (Figure 4).

Previous studies have demonstrated that IL-1β activates NF-κB signaling through p65 phosphorylation in NP cells (Hu et al., 2014; Deng et al., 2018). NF-κB activates its downstream gene targets through multiple mechanisms (Wang et al., 2018; Xu et al., 2018). Specifically, IκBα is preloaded with RNA polymerase, making it rapidly responds to NF-κB binding without requiring time-dependent formation of a preinitiation complex (Ainbinder et al., 2002). In addition, activation of the canonical NF-κB pathway and cytoplasmic release from IκBαcauses RelA Ser^276^ phosphorylation, which is required for the interaction within the P-TEFb complex (Nowak et al., 2008). CDK9, along with cyclin T, is the core constituent of P-TEFb. The P-TEFb complex functions as a transcriptional elongation factor, and participates in the synthesis of mRNAs and the activation of heat shock genes. Moreover, P-TEFb mediates transcriptional elongation by phosphorylating several targets in the paused RNA Pol II-dependent promoter, including small-molecule inhibitors 5, negative elongation factor complex-E, and serine 2 in the largest subunit of RNAPol II (RPB1) C-terminal heptapeptide repeat domain (Palangat et al., 2005).

Diverse signaling pathways can mediate the nuclear phosphorylation of NF-κB subunits and induce the RelA-transactivating subunit to be phosphorylated (Sakurai et al., 1999). NF-κB predominantly exists as a heterodimer between RelA and p50, which binds to the inhibitor IκB protein (Lecoq et al., 2017). Following the stimulation of some cytokines, the two conserved serine residues in the N-terminal domain of IκB become phosphorylated, resulting in its polyubiquitination and subsequent degradation (Barboric et al., 2001). A previous study has demonstrated that Ser^276^ phosphorylation is required for the interaction with the P-TEFb complex. It is further suggested that transcriptional elongation can be mediated by phospho-Ser^276^ RelA binding through recruiting P-TEFb as an additional mechanism for inducible gene expression (Nowak et al., 2008). Furthermore, NF-κB translocates to the nucleus and triggers the innate and adaptive immune response (Miao et al., 2020). These data indicate that NF-κB can activate the subnetworks of target genes controlled by a phosphorylation code (Nowak et al., 2008). Based on other studies, CDK9 inhibitor could suppress the activation of Akt (protein kinase B) associated with the activation of IKK-α. Besides, it has been investigated that IKK is required for cytokine-induced phosphorylation of NF-κB (Ghosh and Karin, 2002). Consequently, as the downstream of IKK-α, the phosphorylation and nuclear translocation of p65 was decreased (Takada and Aggarwal, 2004). In our study, we showed that p-p65 expression was decreased after atuveciclib treatment, indicating that atuveciclib could exert anti-inflammatory effects via the same pathway in NP cells which requires further study. Since atuveciclib treatment also suppressed the function of P-TEFb by inhibiting CDK9 in the nucleoplasm (Figure 5), transcriptional elongation mediated by phospho-Ser^276^ RelA binding P-TEFb could also be suppressed. Moreover, although CDK9 was known as a nuclear kinase that functions in the nucleoplasm, it has been observed that a part of P-TEFb is released from the cytoplasmic pool and enters the nucleus to sustain a high-level of transcriptional activation (Ramanathan et al., 1999; Faust et al., 2018). An *in vivo* assay further confirmed these results (Figure 6).

However, this study has several limitations. Firstly, IVDD is a complex process that cannot be completely simulated using IL-1β treatment. Secondly, the detailed mechanisms by which RNA Pol II suppresses the inflammatory response were not investigated and should be further examined. In addition, our study did not involve other components of IVD, such as adjacent vertebral endplates and annulus fibrosis.

## Conclusion

For the first time, we investigated the role of CDK9 in IVDD progression and the detailed mechanism by which atuveciclib protects against ECM degradation. Since atuveciclib is undergoing clinical trials and has been shown to be orally effective, it may be used as a therapeutic agent for the treatment of inflammatory cytokine-related IVDD in the near future.

## Data Availability Statement

The raw data supporting the conclusions of this article will be made available by the authors, without undue reservation.

## Ethics Statement

The studies involving human participants were reviewed and approved by Ethics Committee of Sir Run Run Shaw Hospital. The patients/participants provided their written informed consent to participate in this study. The animal study was reviewed and approved by Ethics Committee of Sir Run Run Shaw Hospital.

## Author Contributions

ZH and SF designed the experiments. WN, FZ, LZ, YL, and YD performed the experiments and acquired the data. WN, LW, JY, and DH analyzed the data. SF, JY, and ZH supervised the project and wrote the manuscript. All authors contributed to the article and approved the submitted version.

## Conflict of Interest

The authors declare that the research was conducted in the absence of any commercial or financial relationships that could be construed as a potential conflict of interest.
