# Supplementary material for: Cyclin-Dependent Kinase 9 (CDK9) Inhibitor Atuveciclib Suppresses Intervertebral Disk Degeneration via the Inhibition of the NF-κB Signaling Pathway
Source: Front Cell Dev Biol. 2020 Sep 10;8:579658. doi: 10.3389/fcell.2020.579658 (PMC7511812; doi:10.3389/fcell.2020.579658)
Supplement: Supplementary file 1 [file Table_1.DOCX]

Supplementary Table S1: Definition of a Histological Grading Scale

|  | Grade |
| --- | --- |
| I. Anulus fibrosus | 1. Normal, pattern of fibrocartilage lamellae (U-shaped in the posterior aspect and slightly convex in the anterior aspect) without ruptured fibers and without a serpentine appearance anywhere within the anulus  2. Ruptured or serpentined patterned fibers in less than 30% of the anulus  3. Ruptured or serpentined patterned fibers in more than 30% of the anulus |
| II. Border between the anulus fibrosus and nucleus pulposus | 1. Normal  2. Minimally interrupted  3. Moderate/severe interruption |
| III. Cellularity of the nucleus pulposus | 1. Normal cellularity with large vacuoles in the gelatinous structure of the matrix  2. Slight decrease in the number of cells and fewer vacuoles  3. Moderate/severe decrease (>50%) in the number of cells and no vacuoles |
| IV. Matrix of the nucleus pulposus | 1. Normal gelatinous appearance  2. Slight condensation of the extracellular matrix  3. Moderate/severe condensation of the extracellular matrix |

Grades ranged from 4 to 12, where normal is 1 point for each of the 4 categories listed above, for a total of 4 points (Grade 4). Because there is a maximum of 3 points for each parameter, a total of 12 points (Grade 12) is representative of severe degeneration.

| Supplementary Table S2: Primers and sequences used in this study | | |
| --- | --- | --- |
| Primers for qPCR | | |
| Gene | Forward primer: | Reverse primer |
| Human ADAMTS5 | GGGCACTGGCTACTATGTGG | CGTCACAGCCAGTTCTCACA |
| Human Aggrecan | CTACCAGTGGATCGGCCTGAA | CGTGCCAGATCATCACCACA |
| Human Collagen2 | ATGACAATCTGGCTCCCAAC | GAACCTGCTATTGCCCTC |
| Human CDK9 | ATGGCAAAGCAGTACGACTCG | GCAAGGCTGTAATGGGGAAC |
| Human MMP3 | CCTACAAGGAGGCAGGCAAG | CCCGTCACCTCCAATCCAAG |
| Human MMP13 | TCGGCCACTCCTTAGGTCTT | AAGTGGCTTTTGCCGGTGTA |
| Human β-actin | AGAGCTACGAGCTGCCTGAC | AGCACTGTGTTGGCGTACAG |
| Human iNOS | ATCTTCGCCACCAAGCAG | CGACCTGATGTTGCCATTGT |
| Rat ADAMTS5 | GCAGTGGGAGGAAACACAG | CCGCCAGAGTAGAGTTGGTC |
| Rat Aggrecan | CAGTGCGATGCAGGCTGGCT | CCTCCGGCACTCGTTGGCTG |
| Rat Collagen2 | CTGGAAAAGCTGGTGAAAGG | GGCCTGGATAACCTCTGTGA |
| Rat CDK9 | CCTCCGGCACTCGTTGGCTG | GATTTCGGCTCTGGTTGGT |
| Rat MMP3 | TGATGAACGATGGACAGATGA | AGCATTGGCTGAGTGAAAGAG |
| Rat MMP13 | ACTGAGAGGCTCCGAGAAATG | GAACCCCGCATCTTGGCTT |
| Rat β-actin | CTATGAGGGTTACGCGCTCC | ATGTCACGCACGATTTCCCT |
| Rat iNOS | AGGTGCTATTCCCAGCCCAA | GGGTCGATGGAGTCACATGC |
| SiRNAs and miRNAs | | |
| Human CDK9 si | GGCCAAACGTGGACAACTA | |
| Rat CDK9 si | CAACCTGATTGAGATTTGT | |
|  |  | |
